# Endoplasmic reticulum stress promotes hepatocellular carcinoma by modulating immunity: a study based on artificial neural networks and single-cell sequencing

**DOI:** 10.1186/s12967-024-05460-9

**Published:** 2024-07-15

**Authors:** Zhaorui Cheng, Shuangmei Li, Shujun Yang, Huibao Long, Haidong Wu, Xuxiang Chen, Xiaoping Cheng, Tong Wang

**Affiliations:** 1 Department of Emergency, The Eighth Affiliated Hospital of Sun Yat- sen University, Shenzhen, 518003 Guangdong P. R. China; 2 The First Affiliated Hospital of Jiangxi Medical College, Nanchang University, Nanchang, 330000 Jiangxi P. R. China

**Keywords:** Hepatocellular carcinoma, Endoplasmic reticulum stress, Artificial neural network, Prognosis, Immune infiltration

## Abstract

**Introduction:**

Hepatocellular carcinoma (HCC) is characterized by the complex pathogenesis, limited therapeutic methods, and poor prognosis. Endoplasmic reticulum stress (ERS) plays an important role in the development of HCC, therefore, we still need further study of molecular mechanism of HCC and ERS for early diagnosis and promising treatment targets.

**Method:**

The GEO datasets (GSE25097, GSE62232, and GSE65372) were integrated to identify differentially expressed genes related to HCC (ERSRGs). Random Forest (RF) and Support Vector Machine (SVM) machine learning techniques were applied to screen ERSRGs associated with endoplasmic reticulum stress, and an artificial neural network (ANN) diagnostic prediction model was constructed. The ESTIMATE algorithm was utilized to analyze the correlation between ERSRGs and the immune microenvironment. The potential therapeutic agents for ERSRGs were explored using the Drug Signature Database (DSigDB). The immunological landscape of the ERSRGs central gene PPP1R16A was assessed through single-cell sequencing and cell communication, and its biological function was validated using cytological experiments.

**Results:**

An ANN related to the ERS model was constructed based on SRPX, THBS4, CTH, PPP1R16A, CLGN, and THBS1. The area under the curve (AUC) of the model in the training set was 0.979, and the AUC values in three validation sets were 0.958, 0.936, and 0.970, respectively, indicating high reliability and effectiveness. Spearman correlation analysis suggests that the expression levels of ERSRGs are significantly correlated with immune cell infiltration and immune-related pathways, indicating their potential as important targets for immunotherapy. Mometasone was predicted to be the most promising treatment drug based on its highest binding score. Among the six ERSRGs, PPP1R16A had the highest mutation rate, predominantly copy number mutations, which may be the core gene of the ERSRGs model. Single-cell analysis and cell communication indicated that PPP1R16A is predominantly distributed in liver malignant parenchymal cells and may reshape the tumor microenvironment by enhancing macrophage migration inhibitory factor (MIF)/CD74 + CXCR4 signaling pathways. Functional experiments revealed that after siRNA knockdown, the expression of PPP1R16A was downregulated, which inhibited the proliferation, migration, and invasion capabilities of HCCLM3 and Hep3B cells in vitro.

**Conclusion:**

The consensus of various machine learning algorithms and artificial intelligence neural networks has established a novel predictive model for the diagnosis of liver cancer associated with ERS. This study offers a new direction for the diagnosis and treatment of HCC.

## Introduction

As one of the most commonly primary malignancies, liver cancer has become one of the top five causes of cancer-related death around the world according to the World Health Organization [1]. Approximately 90% of liver cancer patients die from hepatocellular carcinoma (HCC) which is the most common pathological type [2]. Surgical treatment remains the most effective way to HCC. However, due to the insidious onset and rapid progression of HCC, patients frequently fail to avail themselves of the surgical option because of delayed medical consultation [3]. Moreover, HCC patients often face the daunting challenge of high prevalence in chemotherapy drug resistance, distant metastasis and recurrences, consequently resulting in an unfavorable prognosis [3, 4]. Therefore, it is vital to deeply investigate the underlying mechanism of HCC occurrence and development, so that we can find new and promising targets for diagnosis and treatment of HCC patients.

Endoplasmic reticulum (ER) involved in lipid and carbohydrate metabolism and calcium strorage [5, 6]. Moreover, as the largest and the most powerful organelle in eukaryotic cells, ER is also mainly responsible for the synthesis, transportation and folding of protein [5, 6]. Endoplasmic reticulum stress (ERS) refers to the protein folding disorder in ER under pathological or physiological stimuli, such as activation of oncogenes, oxidative stress, hypoxia, and infection [7, 8]. ERS regulate three main pathway of unfolded protein response (UPR), including PRKR-like ER kinase, activated transcription factor 6, and inositol requirement Enzyme 1 which alleviate the load of unfolded proteins load, and maintain cell homeostasis and function [9]. UPR pathways are activated in most cancer types because protein synthesis increases dramatically during the rapid proliferation of tumor cells [10, 11]. As the initiating factor of UPR, ERS plays a crucial role in the therapy response and prognosis of cancer. At the beginning of chemotherapy, drugs cause deficiencies in nutrients and hypoxia of tumor cells, which lead to the ERS followed by UPR [12, 13]. Once the UPR is activated, tumor cells release pro-survival components including cytokines, growth factors, and other factors, which induce cancer cell growth and proliferation and suppressing anti-tumor immune response [14, 15], It is reported that when HCC mice were treated with the IRE1α-inhibitor, alleviation of tumor load and collagen accumulation were observed, which indicate that regulating ERS and UPR is an effective way to inhibit drug resistance to HCC.

In our study, machine learning techniques such as Random Forest (RF) and Support Vector Machine (SVM) algorithms were applied to screen for key genes associated with hepatocellular carcinoma (HCC). Subsequently, by integrating these genes, an artificial neural network was utilized to construct an ERS-related HCC diagnostic model. On the training set and three validation sets, the diagnostic model exhibited satisfactory predictive performance. We also conducted a comprehensive analysis of the expression levels, immune infiltration, methylation, and mutation status of ERSRGs. Our research offers a novel perspective on understanding the molecular mechanisms of HCC and identifies potential targets for developing new diagnostic and therapeutic strategies for HCC.

## Methods

### Data sources used for analysis

**Fig. 1:**
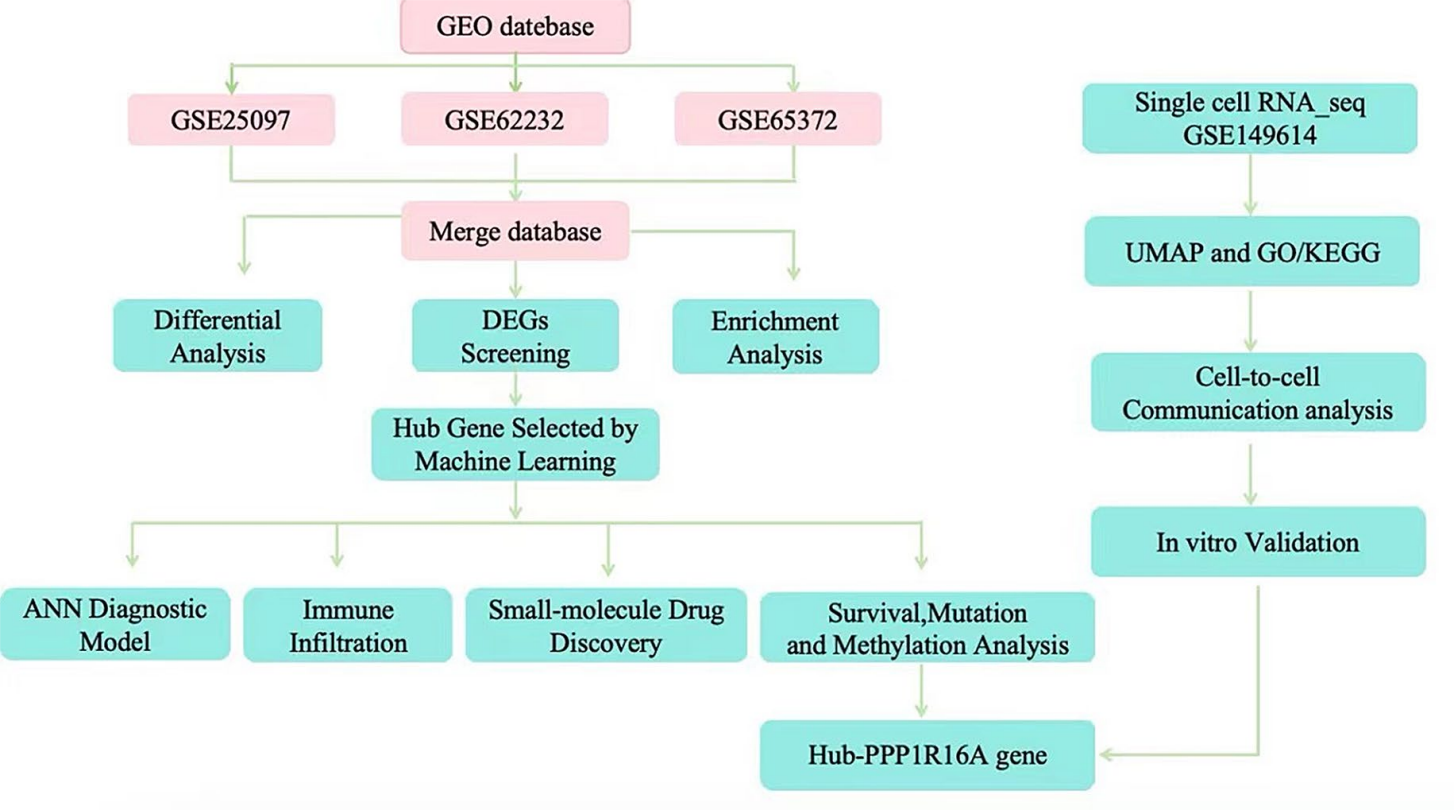
The overall flow of this study

The author first integrated gene expression matrices from GSE25097, GSE62232, and GSE65372, analyzed the gene expression differences between normal and liver cancer tissues, and conducted functional enrichment analysis. By comparing the intersection of differentially expressed genes with genes related to endoplasmic reticulum stress, and employing two machine learning methods, six candidate biomarkers were identified, including SRPX, THBS4, CTH, PPP1R16A, CLGN, and THBS1. Based on these genes, an artificial neural network (ANN) algorithm was utilized to construct a diagnostic model. Subsequently, the diagnostic performance of these candidate genes was validated in three independent validation sets (GSE121248, GSE45267, and GSE84005). Moreover, molecular docking was employed to screen potential target drugs, and the immune cell infiltration rate, methylation level, and mutation rate of the marker genes were assessed. It was found that PPP1R16A exhibited a high copy mutation rate and was significantly correlated with the level of immune cell infiltration. To further identify PPP1R16A as a core gene in the endoplasmic reticulum stress model, single-cell sequencing and cell communication analyses were conducted to study its expression and distribution patterns in the tumor microenvironment. Finally, the biological function of the PPP1R16A gene was validated through in vitro experiments. The overall design of this study is illustrated in Fig. 1.

### Data collection and preprocessing

Transcriptome data and clinical information of HCC patients and normal tissue donors were from the GEO database (https://www.ncbi.nlm.nih.gov/geo/). Three datasets were obtained, including GSE25097 (249 normal tissues and 268 tumor tissues), GSE62232 (10 normal tissues and 81 tumor tissues), and GSE65372 (15 normal tissues and 39 tumor tissues). These datasets were combined and removed repeating tissues using “sva” R package. A total of 662 samples were obtained, and the expression matrix of 14,738 genes was used as the training set. Besides, validation sets consisted of three datasets including GSE121248 (37 normal tissues and 70 tumor tissues), GSE45267 (39 normal tissues and 48 tumor tissues), and GSE84005 (38 normal tissues and 38 tumor tissues). All data is standardized and log-transformed by using the R “limma” software package for subsequent analysis [16].

### The identification of differential expressed genes and ERSRGs

The “limma” R package was used to detect differential expressed genes (DEGs) between HCC and normal tissues in the training set with |log2 fold change (FC)|> 1.5 and adjusted *p* < 0.05 as cutoff value. The volcano plot and heat map showing the differential expression of genes between HCC and normal tissue were made using the “ggplot2” and “heatmap” R packages. The Gene Ontology (GO) and Kyoto Encyclopedia of Genes and Genomes (KEGG) functional enrichment analysis were conducted among DEGs using “clustersProfiler”, “enrichplot”, “limma”, “ggplot2” and “org.Hs.eg.db” R package. The hallmark gene set “h.all.v7.4.symbols.gmt” was downloaded from MSigDB datasets (https://www.gsea-msigdb.org/)^17^ and used for gene set variance analysis (GSVA) analysis with the *p* < 0.05 and false discovery rate (FDR) < 0.25. In addition, 15 hallmark gene sets including 312 ERSRGs were also downloaded from the MSigDB database.

### The construction and validation of artificial neural network (ANN) prediction model using artificial intelligence algorithms

With “random Forest” R package, we established the RF model using fivefold cross-validation method to iterate on the variables’ number at each split and tree. When the number of branches was 125, we got the minimum residual error. We ranked genes according to Gini coefficient score and those genes with score > 20 were finally selected [18, 19]. Using the “e1071” and “caret” R package, the SVM algorithm is applied to delete the SVM-generated feature vectors and identify the optimal variables. We fitted a linear SVM model, sorted the variables by their weights and eliminated the variables with low weights. Through the cross validation, the number of selected genes was determined when root mean square error is minimal [20]. ERSRG with RF and SVM algorithms, the ANN predictive model was established. The ANN model was constructed based on a multilayer perceptron network using R package “neuralnet” and “NeuralNetTools”. This ANN model include input layers, hidden layers, and output layers, and was tested using back-propagation algorithms. The first layer through input layers, neurons transmit the weighted data to neural groups, and then the hidden layers was applied to randomly select bias. Once the hidden nodes’ net sums is validated, the output responses were provided through transfer function [21]. In our study, six ERSRG were selected as the input nodes, as well as HCC and normal tissues were used as the output nodes. Besides, the predictive performance of ANN prognostic models was evaluated through the area under curve (AUC) of time-dependent receiver operating characteristics (ROC) curves using R package “pROC”.

### Survival and methylation analysis of HCC patients

Survival analysis for six ERSRG was performed using the gene expression profiling interactive analysis (GEPIA) website (http://gepia.cancer-pku.cn/index.html). The Kaplan–Meier (K-M) survival analysis was performed to compare patient differences between high and low expression of ERSRG group in conjunction with the log-rank test. Obtain the methylation levels of the six ERSRGs from the UALCAN website (https://ualcan.path.uab.edu/)

### Immune, co-mutation and genetic alterations analysis of six ERSRG in HCC patients

The infiltration of 29 type of immune cells and immune-related pathways were analyzed in HCC patients through ssGSEA analysis using the “GSVA” and “GSEABase” R package [22, 23]. The relationship of ERSRG expression with immune cell infiltration and pathway enrichment was identified through Spearman analysis for coefficient calculation. Genomic data of six ERSRG including somatic mutations and DNA copy-number alterations was obtained from cBioPortal website (https://www.cbioportal.org/). Besides, co-mutation analysis of six ERSRG was applied with “corplot” R package to explore the expression correlation among them.

### Single-cell RNA-seq analysis

GSE149614, liver cancer single cell data set, which includes 10 liver cancer samples. Seurat package (version 4) is used for single cell data processing and analysis. Specifically, 3,4411 cells are obtained by preliminary screening according to the data of genes expressed in at least 3 cells and cells expressing at least 200 genes. Further, according to the secondary screening conditions of 500 < nfeature < 5000 and mitochondrial gene ratio < 10%, cell populations retained in each sample is shown in Table 1.

**Table 1:** Cell populations retained in each sample of single-cell RNA-seq analysis

Cancer sample	HCC01T	HCC02T	HCC03T	HCC04T	HCC05T	HCC06T	HCC07T	HCC08T	HCC09T	HCC10T
Cell population	2720	3352	4269	2117	2652	4086	460	4058	2446	2494

Normalize data + find variable features + scale data function pipeline was used to standardize and normalize the data, and runpca function was used to calculate the top 50 principal components. According to the results of jackstraw and elbow plot, the top 20 principal components are the most appropriate. The find clusters function identifies 31 clusters with a resolution of 0.4 to 30, and umap performs dimensionality reduction clustering.

### Cell-cell interaction analysis

The cell communication was analyzed using the R package ‘cell chat’. First, according to the expression of the protein phosphatase 1 regulatory subunit 16 A (PPP1R16A), the presence or absence of hepatocytes was divided into PPP1R16A positive and negative groups, and they were analyzed together with other cells. Additionally, the netAnalysis_compute Centrality function compared the outgoing signals and incoming signals among different cells to determine the core pathways mediating cell-cell interactions, and the hierarchy plot visualization was performed for some selected pathways. The netAnalysis_contribution function calculated the contribution of the receptor-ligand pairs in specific pathways and displayed the ligand-receptor pairs with the highest contribution.

### Cell culture and transfection

The liver cancer cell lines HCCLM3, HepG2, Hep3B, and the normal liver cell line LO2 were all purchased from Shanghai Fuheng Cell Biology Co., Ltd. and cultured according to standard procedures. Lipid-mediated siRNA transfection was performed using the lipo3000 reagent (Invitrogen, USA) according to the siRNA product instructions (Ribio, China). The siRNA sequences targeting PPP1R16A are as follows: siRNA#1 - TGCCCGAAATGACCTGGAA; siRNA#2 - TGCGGCATCTATACTCCAA; siRNA#3 - CCAACATCAATGCCTGTGA.

### Real-time quantitative qRT-PCR

Total RNA from HCC cells was extracted using the TRIzol reagent (Invitrogen) and reverse transcribed into cDNA using PrimeScript Reverse Transcriptase (Takara, Japan) before qRT-PCR. Quantitative PCR was performed using SYBR Premix EX TaqTM II (Takara, Japan) and the LightCycler 480 real-time PCR system (Roche, Shanghai, China). GAPDH was used as an endogenous control gene to normalize the expression of the target gene. Each sample was analyzed in triplicate. The thermal cycling program included holding for 10 s at 95 °C, 30 s at 60 °C, and 60 s at 72 °C. Then, melt curve data was collected. The primer sequences are shown in Table 2.

**Table 2:** Primer sequences

	Primer sequences
PPP1R16A-F	5’-TTGATGATTTCCGAGAGATGGTGC-3’
PPP1R16A-R	5’-GTGTTGACCGCCAGGAGATTG-3’
GAPDH-F	5’-ATCCCTCCAAAATCAAGTGGGG-3’
GAPDH-R	5’-GGGCAGAGATGATGACCCTTTT-3’

### Cell proliferation, migration and invasion assay

After transfecting cells with siRNA for 48 h, 3,000 cells per well were seeded into 96-well plates. Each well was contained 100 µl complete growth medium and 10µL CCK-8 was added and mixed in each well. After 2 h, the absorbance at 450 nm was measured. Cell migration and invasion were determined using a Transwell chamber. In the migration assay, cells (5*10^3^) after 48 h of siRNA transfection were seeded into the upper chamber (Corning) of serum-free medium, and the lower chamber was filled with medium containing 20% FBS. After approximately 24 h, they were fixed with 4% paraformaldehyde for 20 min and stained with 0.1% crystal violet for 15 min. The invasion assay followed the same procedure as the migration assay, except that the lower chamber of the invasion experiment was coated with Matrigel (BD Biosciences).

### Drug prediction and molecular docking

The drug prediction for the core genes of the endoplasmic reticulum stress model was conducted using the DSigDB database from Enrichr (https://maayanlab.cloud/Enrichr/). Subsequently, the small molecules were docked with the aforementioned central targets using AutoDock Vina (Scripps Research, San Diego, CA). The docking results were evaluated and analyzed using the PLIP system (https://plip-tool.biotec.tu-dresden.de/plip-web/plip/index). Finally, the molecular docking (MD) results in two-dimensional structure were visualized using LIGPLOT software version 4.5.3, and the MD diagrams were generated using PyMOL. Protein structures were obtained from the PDB (https://www.pdb.org/) or AlphaFold (https://alphafold.com/), and drug-related data were retrieved from PubChem (https://pubchem.ncbi.nlm.nih.gov/).

### Cell scratch

Cells were seeded equably in 6-wll plate, and then transfected for 48 h. Approximately 5*10^5^ cells were added to each well, and 10uL needles were used to scrape three horizontal lines on the surface of plate. Following this, the cells were cultured in a 2% serum medium for 24 h. Optical microscopy was used to collect images of cells at after 0 and 24 hours.

### Statistical analysis

Version R 4.1.3 was used for all statistical studies. Differences between two groups were compared through Mann–Whitney test for non-normally distributed variables and unpaired t-test for normally distributed variables. Spearman analysis was used for correlation analysis and coefficient calculation. We analyzed the RT-qPCR results using paired t-tests and drew a Venn diagram using funrich software. P value < 0.05 was defined as statistically significant.

## Results

### The identification of DEGs between HCC and normal tissues

**Fig. 2:**
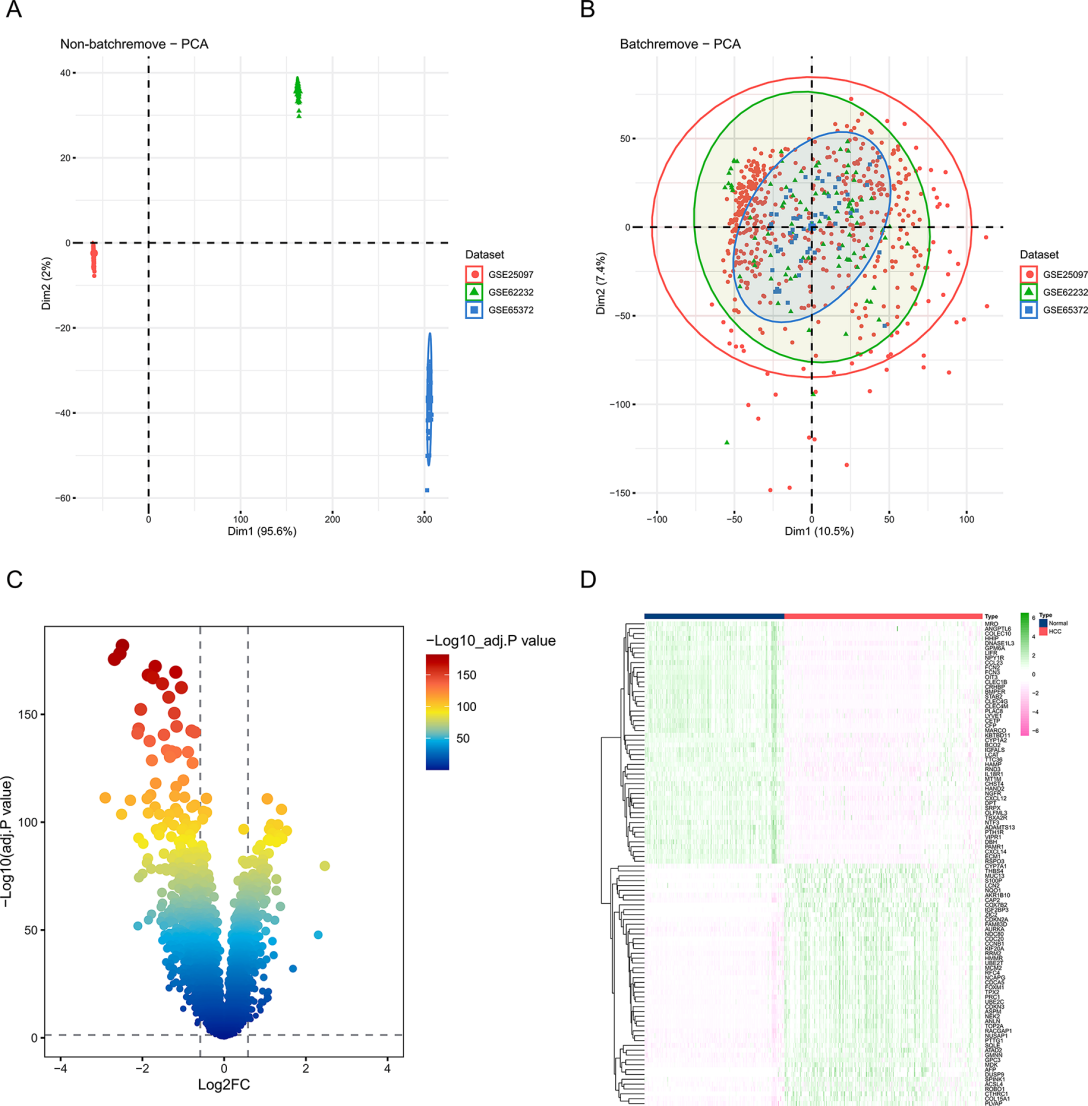
Differential gene expression analysis between liver cancer and normal tissues.(**A**) Principal component analysis (PCA) of genes without batch removal for datasets including GSE25097, GSE62232, and GSE65372. (**B**) PCA of genes with batch removal for datasets including GSE25097, GSE62232, and GSE65372. (**C**) A volcano plot representing 763 differentially expressed genes (DEGs) between liver cancer tissues and normal tissues. (**D**) A heatmap showing the 763 DEGs between HCC and normal tissues

We performed principal component analysis (PCA) of genes of datasets GSE25097, GSE62232 and GSE65372, under both non-batch remove and batch remove condition respectively, and results were presented in Fig. 2A and B. The results show that we successfully removed batch effects from different data sets. The volcano plot in Fig. 2C showed the 763 DEGs between HCC and normal tissues, of which 215 genes were up-regulated and 548 genes were down-regulated. The heatmap in Fig. 2D also presented the expression level of DEGs between HCC and normal tissues.

### GO and KEGG as well as GESA enrichment analysis

**Fig. 3:**
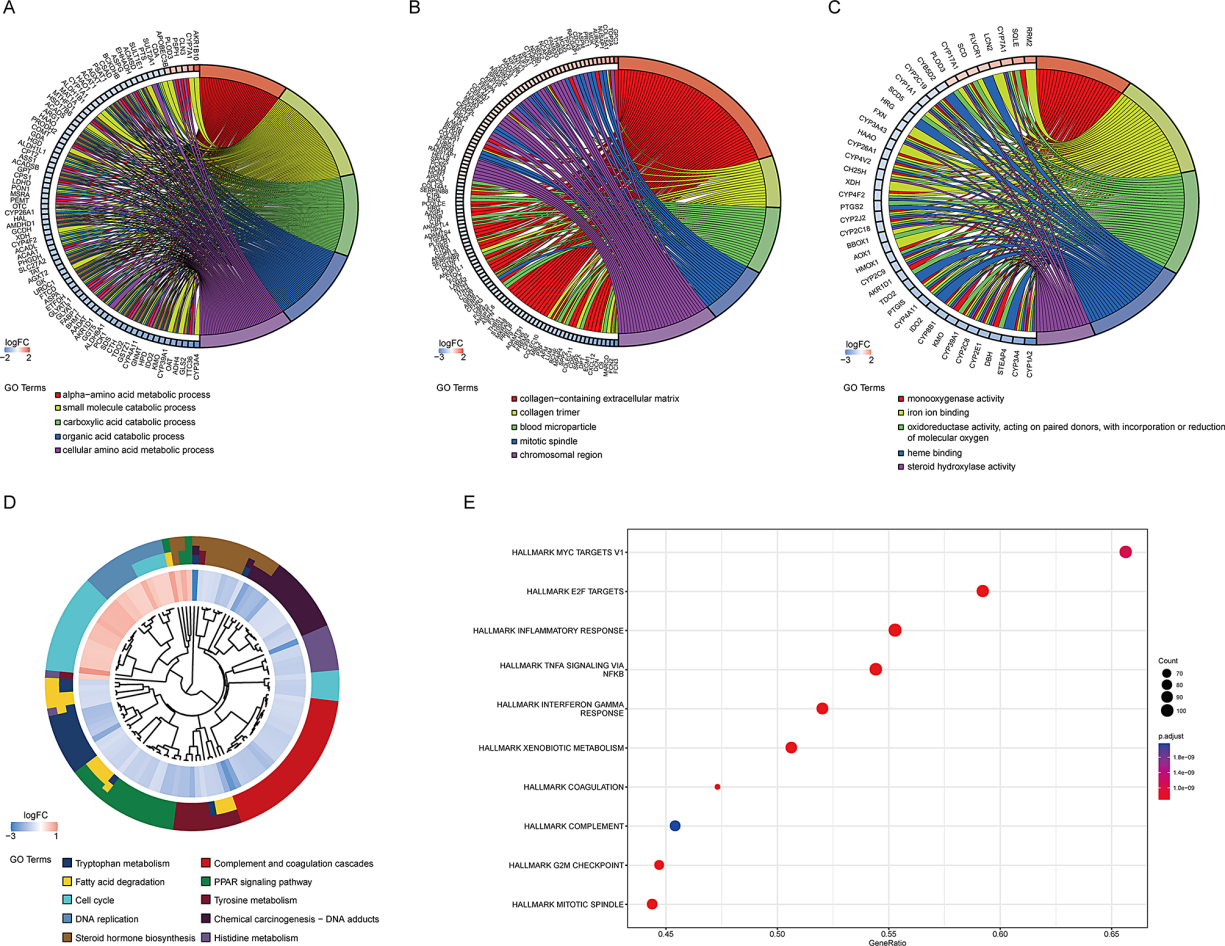
Functional enrichment of DEGs between liver cancer tissues and normal tissues.(**A**-**C**) Gene ontology (GO) analysis of DEGs, including molecular function (MF), cellular component (CC), and biological process. (**D**) Kyoto Encyclopedia of Genes and Genomes (KEGG) analysis of DEGs. (**E**) Gene Set Variation Analysis (GSVA) of DEGs.

The functional enrichment of DEGs between HCC and normal tissues was analyzed. The biological process in GO analysis of DEGs mainly included the alpha-amino acid metabolism, small molecule catabolism, carboxylic acid catabolism, organic acid catabolism, cellular amino acid metabolism. The cellular component in GO analysis was enriched in collagen-containing extracellular matrix, collagen trimers, blood microparticles, mitotic spindle and chromosomal regions. The molecular function in GO analysis was mainly involved in monooxygenase activity, iron ion binding, oxidoreductase activity acting on paired donors, with incorporation or reduction of molecular oxygen, heme binding, and steroid hydroxylase activity (Fig. 3A-C). Besides, the KEGG analysis showed that the DEGs mainly contributed to tryptophan metabolism, complement and coagulation cascades, PPAR signaling pathway, cell cycle, and tyrosine metabolism in Fig. 3D. Furthermore, the GSVA analysis of DEGs was conducted to explore the molecular pathway enrichment of DEGs. The results showed that DEGs were mainly abundant in hallmark MYC targets V1, hallmark E2F targets, hallmark inflammatory response, hallmark TNF-α signal via NF-κB and hallmark interferon-γ response (Fig. 3E).

### The identification of ERS-related DEGs

**Fig. 4:**
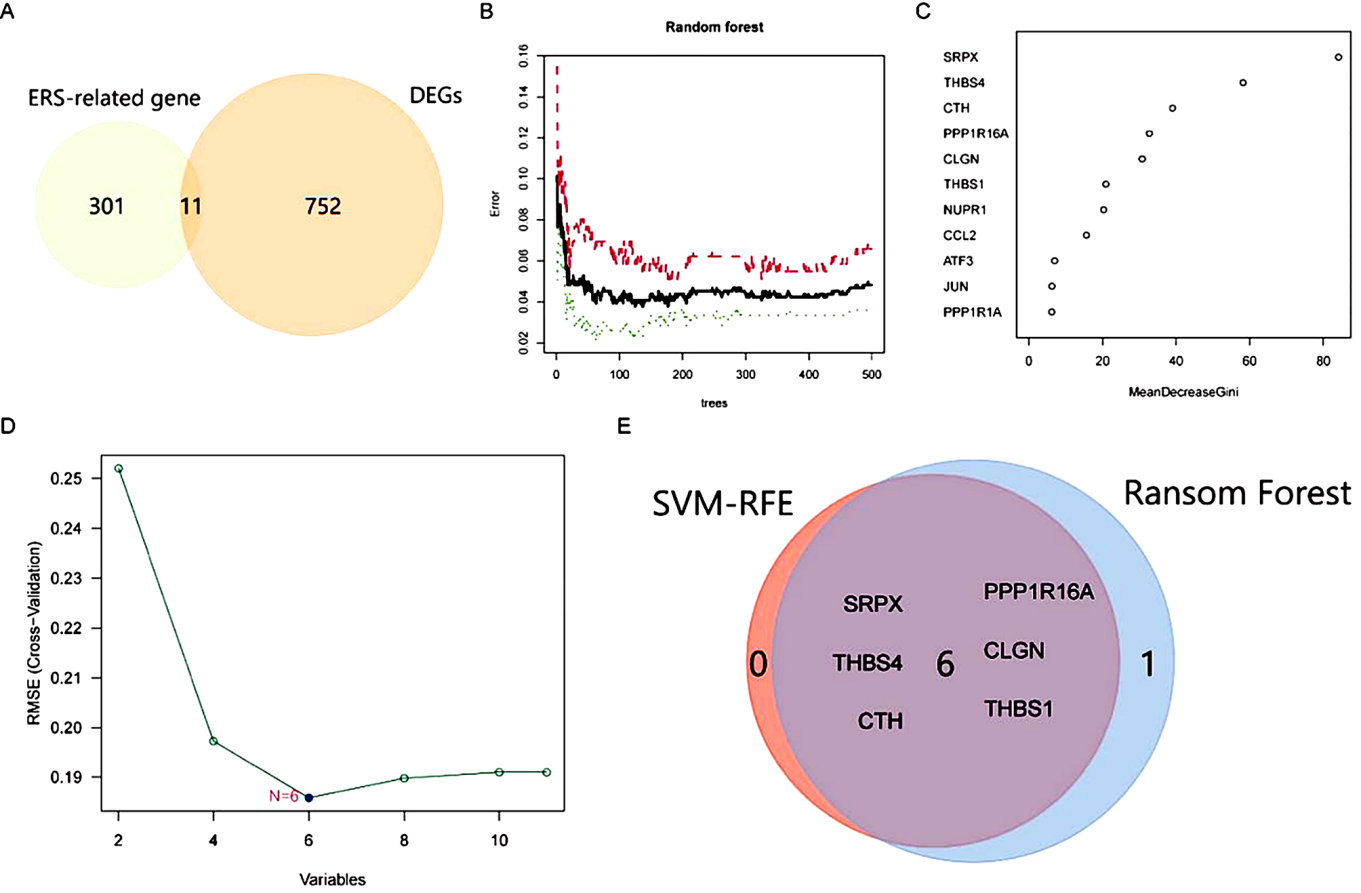
Identification of ERS-related differentially expressed genes. **(A)**ERS-related genes (*n* = 312) were cross-referenced with DEGs (*n* = 763) to identify ERS-related differentially expressed genes (ERSRGs) (*n* = 11). (**B**) Residual plot for the selection of ERSRGs using the Random Forest (RF) algorithm. (**C**) The 11 selected ERSRGs are arranged in descending order according to the Gini coefficient. (**D**) Selection of ERSRGs using the Support Vector Machine (SVM) algorithm. (**E**) Identification of 6 ERSRGs through the intersection of genes selected by RF and SVM.

The 11 ERS-related DEGs were obtained through the intersection of 312 ERSRGs and 763 DEGs in Fig. 4A.A RF analysis further screened 11 ERSRGs. The residual graph in Fig. 4B showed that the residual was the smallest when the number of branches was 125. As presented in Figs. 4C and 11 ERSRGs were scored in this RF tree, and 7 of them with a score of > 20 were finally selected according to the descending Gini coefficient. Additionally, SVM analysis was also applied to screening the 11 ERSRGs. As depicted in Fig. 4D, the root mean square error was the smallest when 6 of 11 ERSRGs were retained. Through the intersection of 7 ERSRGs in RF analysis and 6 ERSRGs in SVM analysis, 6 ERSRGs including SRPX, THBS4, CTH, PPP1R16A, CLGN, and THBS1 were finally determined for the construction of the ANN below (Fig. 4E).

### Construction and validation of ANN prediction model in HCC

**Fig. 5:**
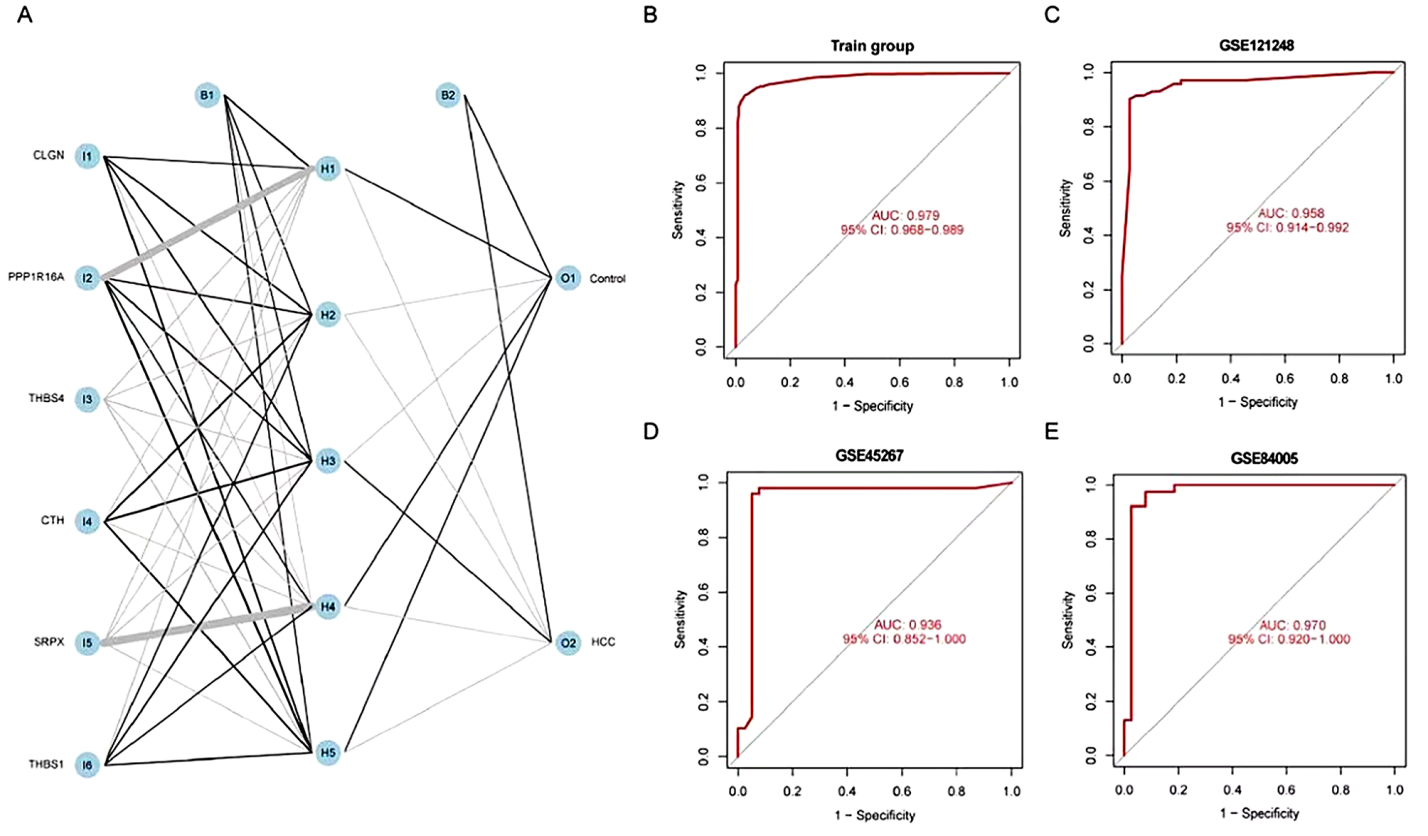
Construction and Validation of the Artificial Neural Network (ANN) Prediction Model for Liver Cancer. (**A**) The ANN comprises 6 neurons as the input layer, 2 neurons as the output layer, and 5 neurons as the hidden layer. (**B**) The AUC value of the ANN prediction model in the training set. (**C**-**E**) The AUC values of the ANN prediction model in the validation groups

The ANN was established including 6 neurons (SRPX, THBS4, CTH, PPP1R16A, CLGN, and THBS1) as the input layer and 2 neurons (HCC and normal tissues, respectively) as the output layer (Fig. 5A). When the hidden layer included 5 neurons, the predictive performance of the model was the highest with the AUC value of 0.979 (95% CI: 0.968–0.989) in the training group (Fig. 5B). The efficiency of this ANN predictive model was also assessed in the other 3 validation groups. The AUC value of the ANN model was 0.958 (95% CI: 0.914–0.992), 0.936 (95% CI: 0.852-1.000) and 0.970 (95% CI: 0.920-1.000) in GSE121248, GSE45267 and GSE84005, respectively (Fig. 5C-E). Above all, this ANN prediction model based on the ER is a potentially powerful tool to predict HCC diagnosis.

### The impact of endoplasmic reticulum stress core genes on immune infiltration deserves attention

**Fig. 6:**
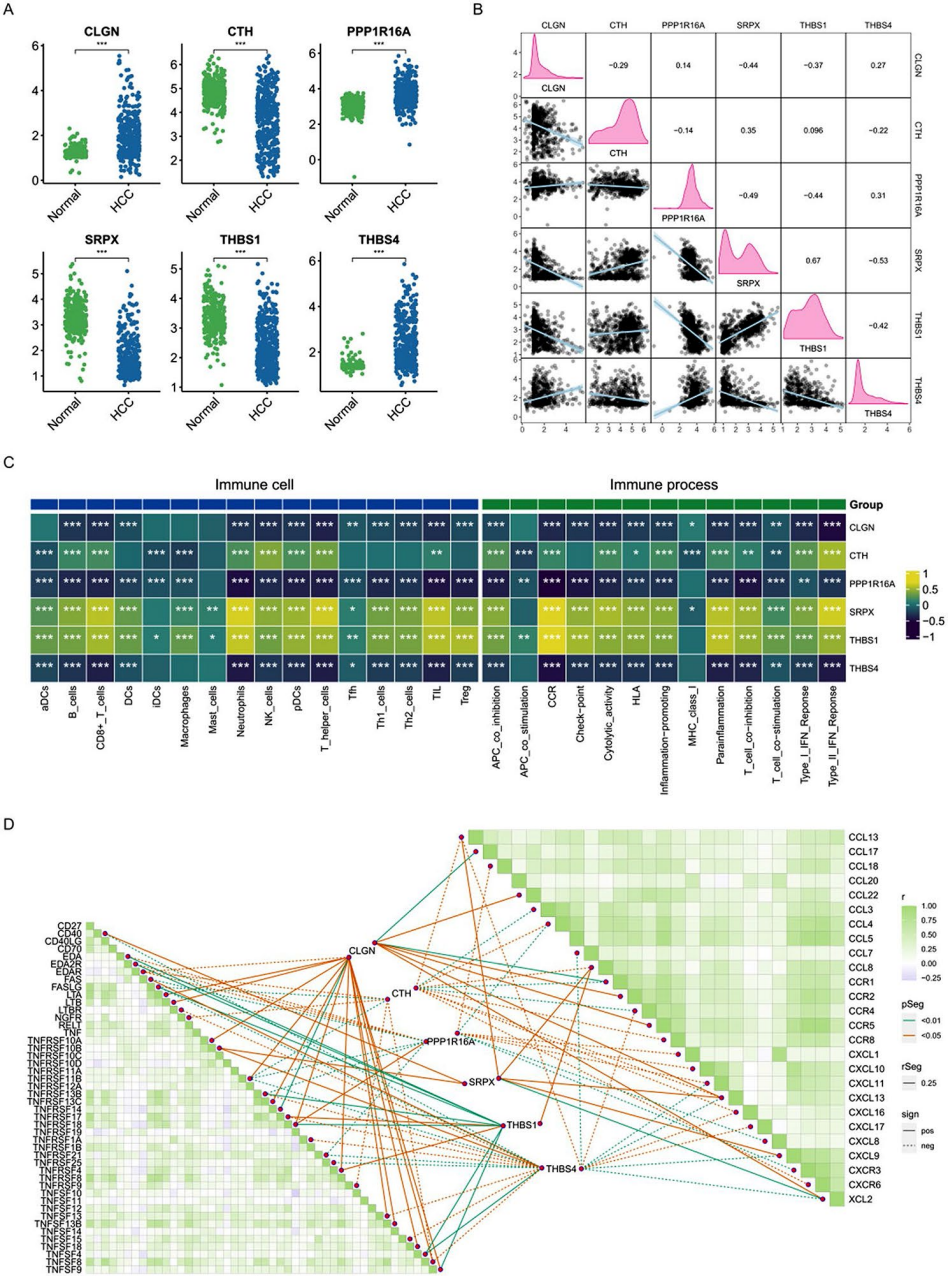
Expression and Immune infiltration Analysis of Six ERSRGs. (**A**) The expression of six ERSRGs between HCC and normal tissues. (**B**) The Co-mutation analysis of six ERSRGs. (**C**) The relationship of six ERSRGs expression levels with the immune cell infiltration and immune-related pathway. (**D**)Correlation between the expression levels of 6 ERSRGs and immune-related molecules

The expression of six ERSRGs between HCC and normal tissues was deeply investigated in Fig. 6A. The results showed that there were significantly higher expression levels of CLGN, PPP1R16A and THBS4 and lower expression levels of CTH, SRPX and THBS1 in HCC tissues compared with normal tissues, implying that CLGN, PPP1R16A and THBS4 as oncogenic genes played an important role in HCC development and progression while CTH, SRPX and THBS1 as onco-suppressor genes may play an inhibitory role in HCC. The interaction of 6 ERSRGs were further analyzed to identify the co-occurrence or mutually exclusive relationship of them in Fig. 6B. There was an intense co-occurrence relationship between THBS1 and SRPX with a correlation coefficient of 0.67 as well as a mutually exclusive relationship between SRPX and THBS4 with a correlation coefficient of -0.53. In order to explore the underlying mechanism that these ERSRGs contributed to HCC occurrence and progression, the relationship of 6 ERSRGs expression levels with the immune cell infiltration and immune-related pathways was analyzed as presented in Fig. 6C. We can conclude that THBS1 and SRPX were significantly positively associated with the infiltration of most immune cells and immune-related pathways such as neutrophils, T helper cells, tumor-infiltrating lymphocytes (TILs), cytokines and chemokines receptors (CCR), and para-inflammation etc. In contrast, THBS4, PPP1R16A and CLGN were significantly negatively associated with the infiltration of most of immune cells and immune related pathways such as neutrophils, T helper cells, TILs, CCR, checkpoint, T cell co-inhibition. Above findings further demonstrated that the ERSRGs can regulate tumor immune micro-environment to expose effects on HCC prognosis.Genes related to the TNF family molecules and chemotactic factors have been collected from previous literature. The ggcor and ggpplot2 packages were utilized to analyze and visualize the expression correlations between core genes and two types of immune activity molecules. The results show green squares representing positive correlations, purple squares indicating negative correlations, solid lines representing positive correlations, and dashed lines depicting negative correlations. Notably, CLGN, SRPX, and THBS1 exhibit positive correlations with most immune activity molecules, whereas PPP1R16A, CTH, and THBS4 show negative correlations with most immune activity molecules (Fig. 6D). This conclusion is largely consistent with the results of the immune cells and processes depicted in the preceding figure.

### Drug screening and molecular docking of characteristic genes

**Fig. 7:**
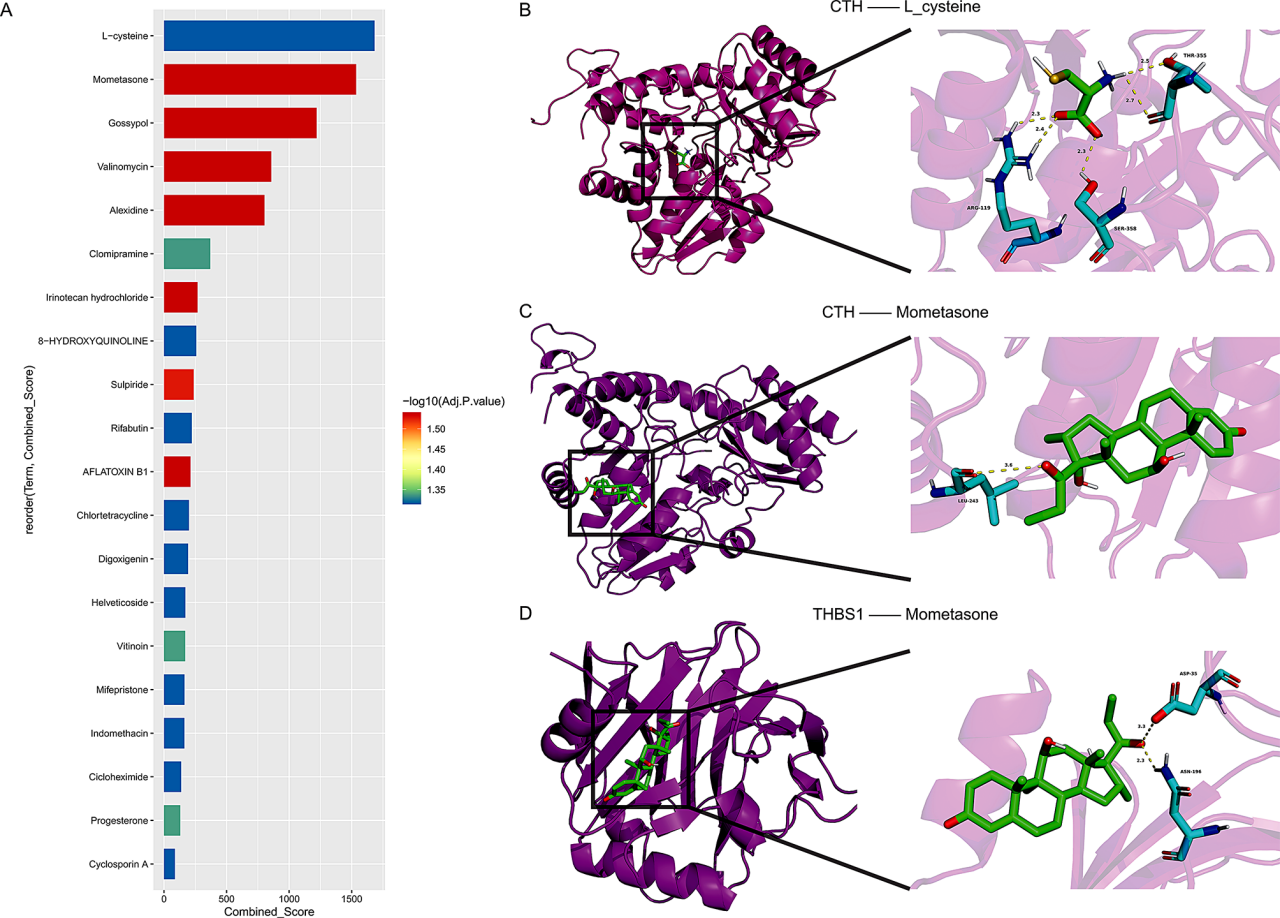
Predict the top 20 candidate drugs for endoplasmic reticulum stress-related genes based on PubChem. (**A**) Predict the top 20 most significant candidate compounds for ERSRGs using the DSigDB database. (**B**-**G**) Molecular docking between THBS1 and Mometasone

The results showed the top 20 drugs with the highest score values, among which L-cysteine had the highest binding score, while Mometasone had the strongest binding significance (Fig. 7A). Therefore, we conducted further molecular docking model simulations of the target genes bound by these two drugs, and the results showed that THBS1 had the lowest binding energy with Mometasone compared to CTH, which was − 6.917 kcal/mol. Therefore, Mometasone may be the most suitable therapeutic drug for THBS1 (Fig. 7B-D).

### The six ERSRGs-related survival, mutation and methylation analysis

**Fig. 8:**
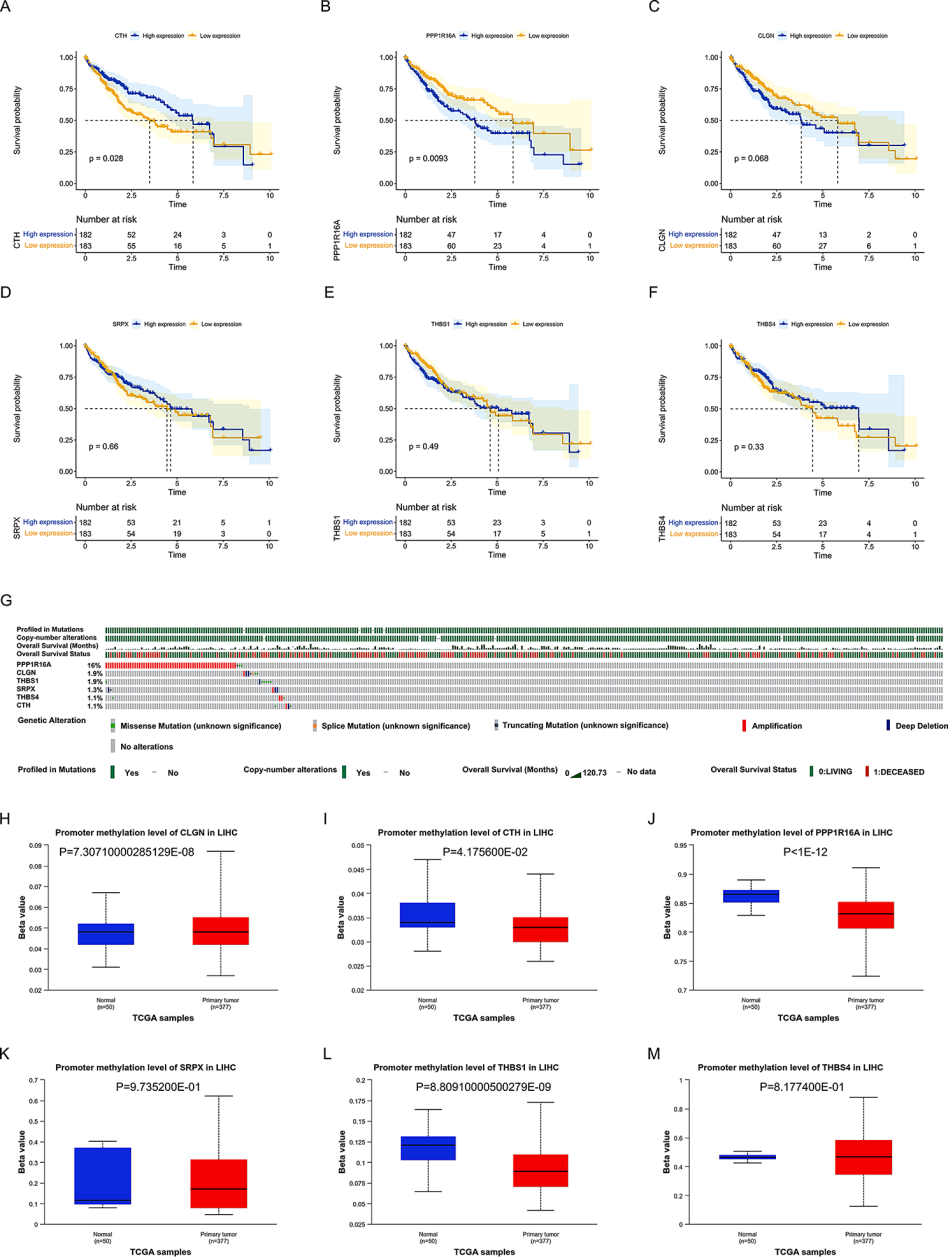
Survival, mutation and methylation Analysis Related to ERSRGs. (**A**-**F**) Kaplan-Meier curves representing the differences in overall survival between groups with high and low expression levels of six ERSRGs. (**G**) Comparison of gene mutations and copy number variations among the six ERSRGs. (**H**-**M**) Calculation of methylation levels of six ERSRGs genes

It was depicted from the Kaplan–Meier (KM) curve that the HCC patients with low expression levels of CLGN and PPP1R16A had significantly longer overall survival than those with high expression levels of two genes (Fig. 8A and C). By contrast, HCC patients with high expression levels of CTH statistically lived longer compared with those with low expression level of CTH (Fig. 8B). However, there was no significant difference in the prognosis of HCC patients between high and low expression of SRPX, THBS1 and THBS4 which needed further validation (Fig. 8D-F). In addition, gene mutation and copy number variation of 6 ERSRGs in 379 HCC patients from the cBioportal website was compared in Fig. 7G. There was the highest mutation rate of PPP1R16A among 6 ERSRGs which was characterized by gene amplification. In addition to describing the survival and mutation status of core ERS genes, we also analyzed the methylation status of the promoters corresponding to these core genes. According to the calculation method on the UALCAN website (https://ualcan.path.uab.edu/), we found that there were significant differences in the degree of promoter methylation between the control group and the liver cancer cell group for CLGN, CTH, PPP1R16A, and THBS1. Among them, the promoter methylation level of CLGN was stronger in the tumor group, while the methylation levels of the other three genes were significantly reduced in the tumor group, especially PPP1R16A (Fig. 8H-M).

### Single-cell sequencing analysis of the immune landscape of the PPP1R16A gene

**Fig. 9:**
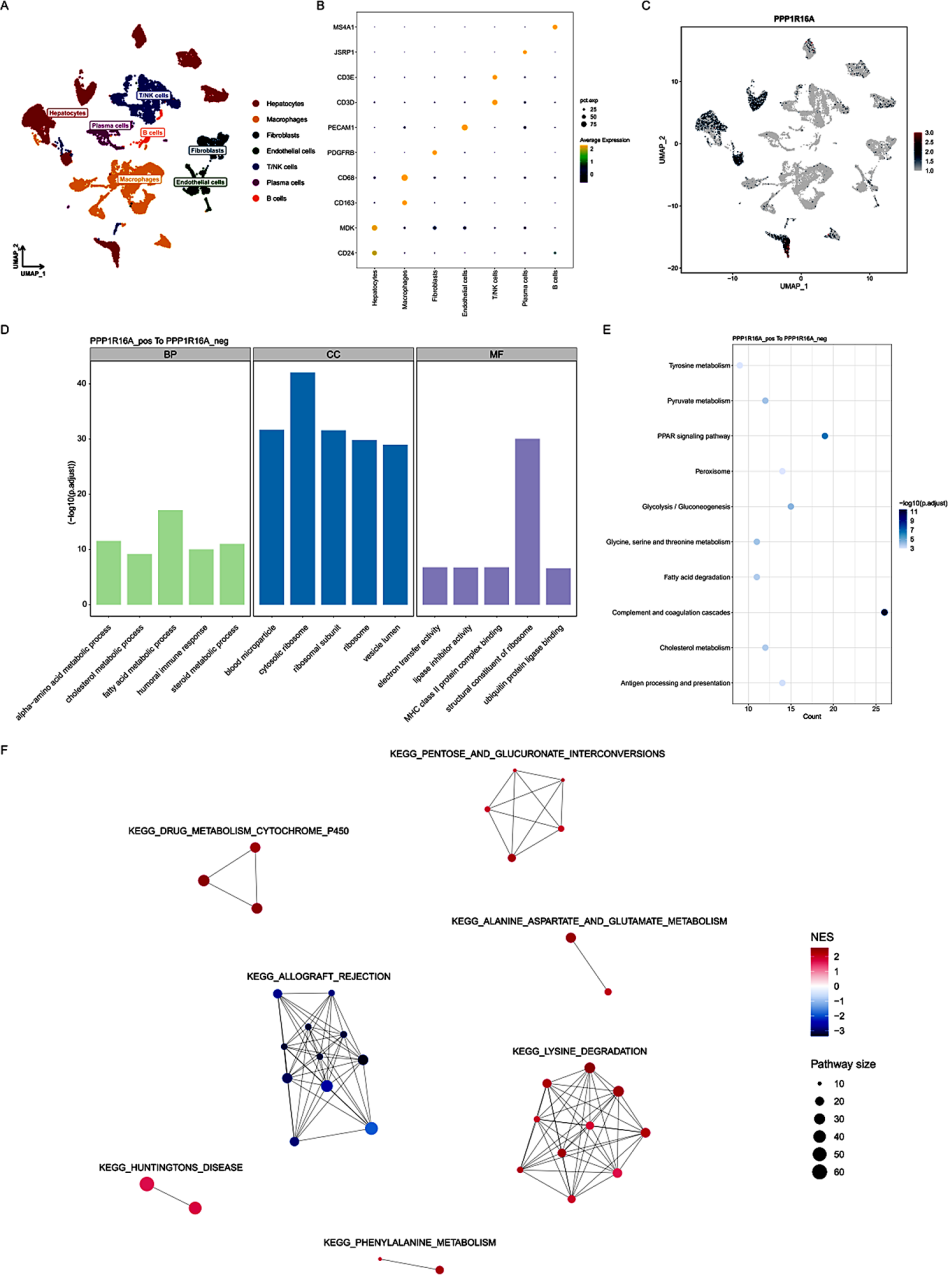
Single-cell RNA-seq Data Analysis (GSE149613). **(A)** UMAP plot of annotated cell types. (**B**) Bubble chart showing markers corresponding to different cell types. (**C**) UMAP diagram shows the expression of ppp1R16A (**D**) Go enrichment analysis, including BP, CC, MF. (**E**)KEGG enrichment analysis; The color close to blue indicates a smaller p value, and the larger bubble table indicates that more differential genes are enriched in this pathway.(**F**)Analysis of downstream signaling pathways of GSEA at the single-cell level

The UMAP diagram shows that all cells can be annotated into seven types of cells, including liver parenchymal cells, macrophages and so on (Fig. 9A). The bubble chart shows the marker genes used in seven different annotation cell groups. Such as liver Parenchymal cells (CD24, MDK), Macrophages (CD68, CD163), Fibroblasts (PDGFRb), Endothelial cells (PECAM1), T/NK cells (CD3D, CD3E), Plasma cells (JSRP1), B cells (MS4A1) (Fig. 9B). Single cell analysis showed that PPP1R16A was mainly expressed in malignant liver parenchymal cells (Fig. 9C). Go enrichment analysis showed that the cells with high expression of PPP1R16A were mainly related to the process of lipid metabolism, such as cholesterol metabolism, fatty acid metabolism and so on (Fig. 9D). KEGG enrichment analysis showed that PPP1R16A was related to glucose metabolism, lipid metabolism and PPAR signaling pathway. These results suggest that PPP1R16A is highly enriched in liver parenchyma, which may aggravate the progression of liver cancer by affecting metabolism related pathways (Fig. 9E).To further explore the downstream pathways of PPP1R16A, we conducted a GSEA analysis of KEGG at the single-cell level. The differential analysis results of all genes were sorted by their logFC, and GSEA analysis was performed using the clusterProfiler package and GSVA package. The resulting KEGG analysis results were further clustered and visualized using the aPEAR package. The results showed that among all significant signaling pathways, seven pathways had clustered modular characteristics and were more core signaling pathways (Fig. 9F).

### Exploration of the impact of the PPP1R16A gene in the tumor microenvironment through cellular communication

**Fig. 10:**
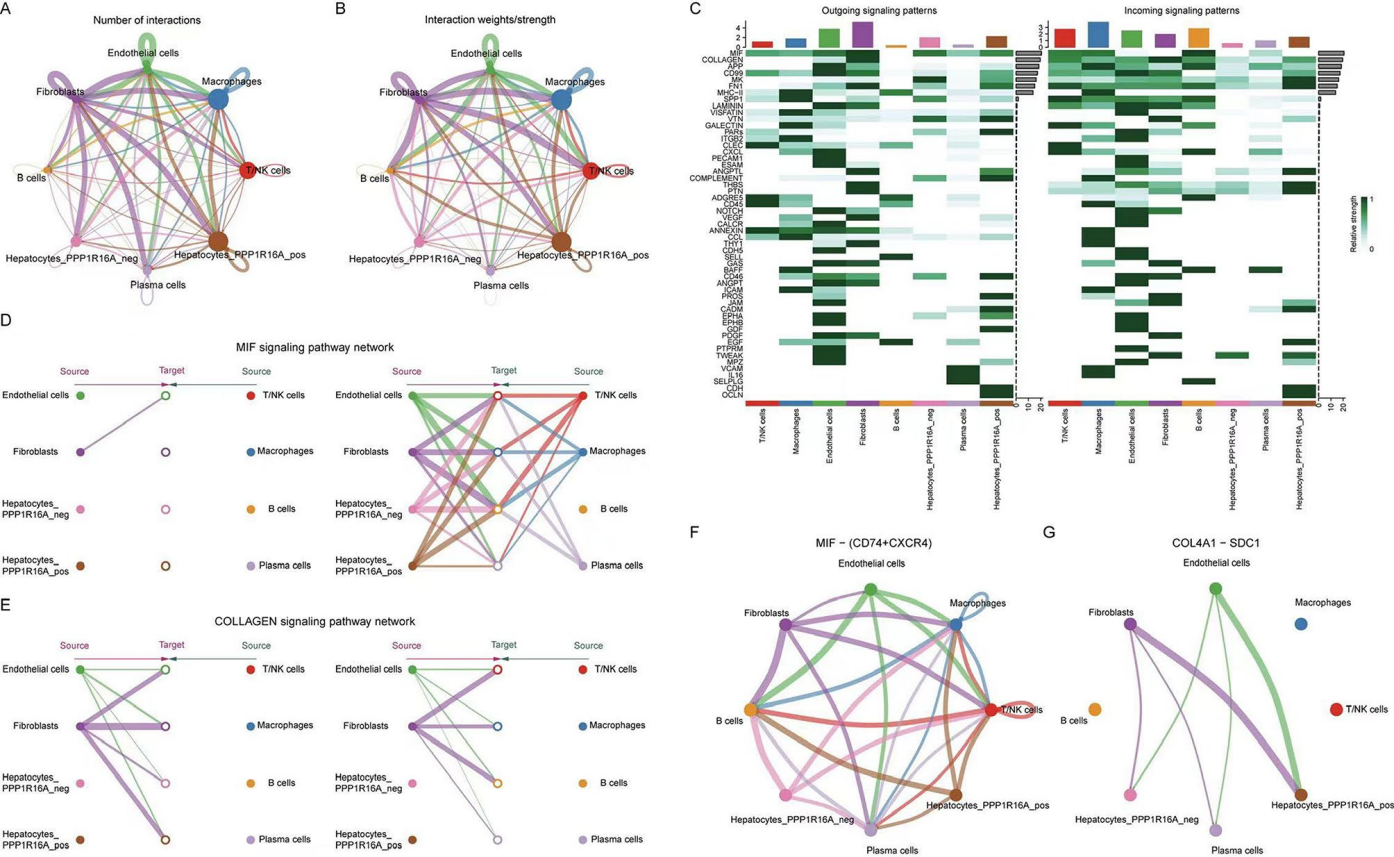
Inference of cell–cell communications in TME. (**A**-**B**) A cell–cell communications between the identified cell types. (**C**) The incoming and outgoing signaling pathways of each cell type. (**D**-**G**) The hierarchical diagram displays the specific interaction between MIF and COLLAGE pathways

Based on the expression of PPP1R16A in malignant liver parenchymal cells, we can divide the liver parenchymal cells into PPP1R16A_pos and PPP1R16A_neg. Using the Cellchat package to calculate the cell communication between these two types of cells and other cell types, we found that fibroblasts, endothelial cells, and PPP1R16A_pos cells are the core cells in the communication network, with fibroblasts being the cell with the strongest output signals. From the perspective of PPP1R16A_pos as the signal sender, it has more communication numbers and intensities with endothelial cells and macrophages (Fig. 10A-B). PPP1R16A_pos cells had more communication with VTN, PARs, complement, CD46, PROS, CADM, GDF, CDH, and OCLN compared to other cells. When looking only at PPP1R16A_pos cells themselves, the strongest output signaling pathway was the macrophage migration inhibitory factor (MIF) pathway. In a lateral comparison, PPP1R16A_pos cells had more communication with MK, FN1, ANGPTL, THBS, PTN, CADM, EGF, CDH, and OCLN. When looking only at PPP1R16A_pos cells themselves, the strongest received signal was the COLLAGEN pathway (Fig. 10C). We further studied the specific interactions between MIF and COLLAGE pathways. Hierarchical diagram shows that PPP1R16A_pos cells, as MIF signal senders, mainly send signals to T/NK cells, macrophages, B cells, and plasma cells (Fig. 10D). As a COLLAGEN signal receiver, it mainly receives signals from endothelial cells and fibroblasts, of which fibroblasts received the most signals (Fig. 10E). Further mining of the ligands and receptors that are most likely to play a role in the pathway, the highest pairing probability in the MIF pathway was between the MIF ligand and the CD74 + CXCR4 receptor (Fig. 10F), and the highest probability of pairing in the COLLAGEN pathway was between the COL4A1 ligand and the SDC1 receptor (Fig. 10G).

### Upregulation of PPP1R16A in liver cancer and knockdown of PPP1R16A significantly suppresses the proliferative, invasive, and migratory abilities of HCC cells

**Fig. 11:**
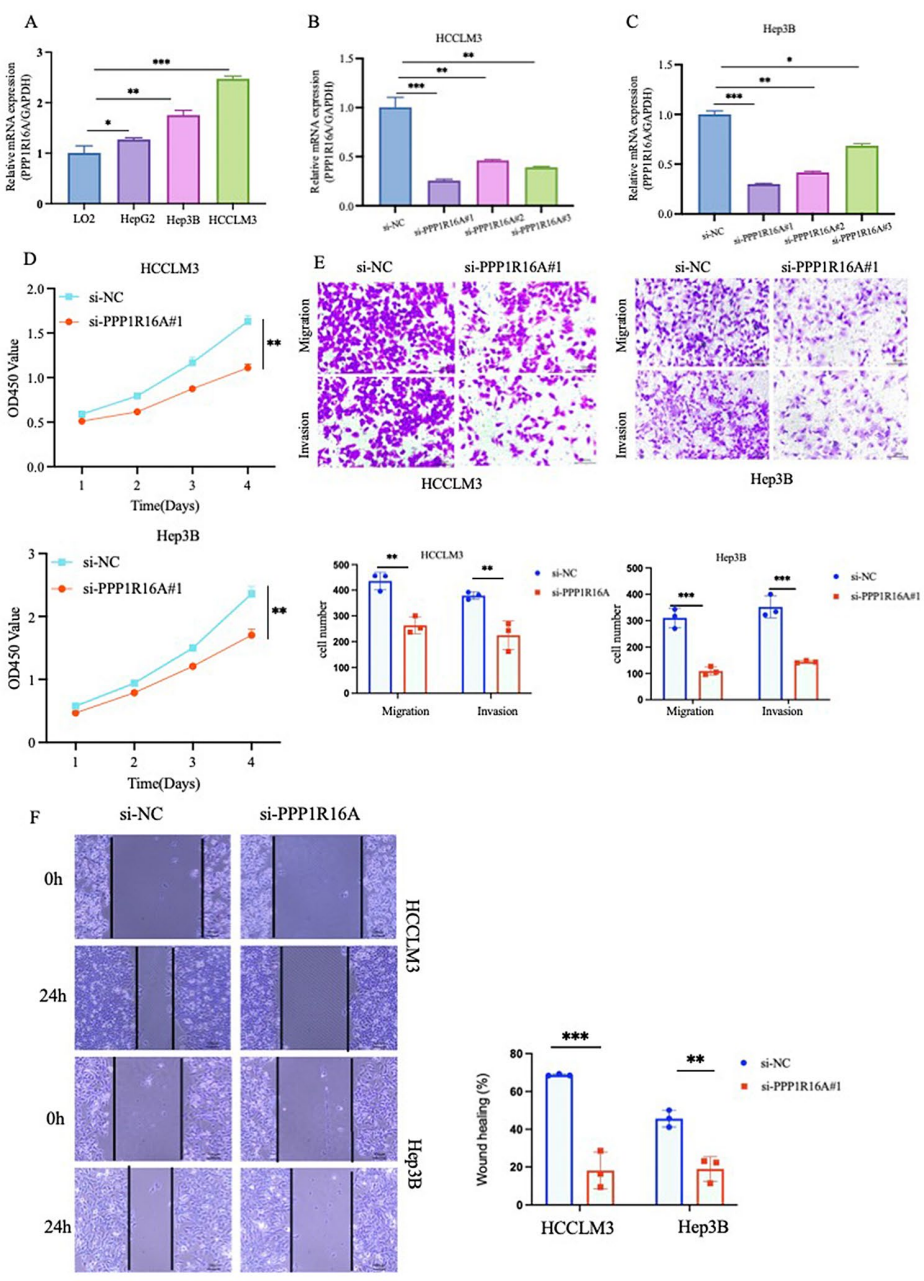
Validation of PPP1R16A Expression Levels and Knockout of PPP1R16A Significantly Inhibits Proliferation, Invasion, and Migration Capabilities of HCC (**A**)Expression levels of PPP1R16A mRNA in HCC cell lines. (**B**-**C**) Knockout efficiency of PPP1R16A mRNA in Hep3B and HCCLM3 cells. (**D**) CCK8 assays indicate that the knockdown of PPP1R16A inhibits the proliferation abilities of Hep3B and HCCLM3 cells. (**E**-**F**) Knockout of PPP1R16A inhibits the migration and invasion capabilities of Hep3B and HCCLM3 cells (*p < 0.05, **p < 0.01, ***p < 0.001, ns: not significant)

By summarizing all the aforementioned findings, we have established that PPP1R16A was a pivotal gene associated with ERS and exhibiting a high copy number mutation. QRT-PCR experiments, showed that human HCC cell lines exhibited a higher expression of PPP1R16A compared to normal liver cells (Fig. 11A). We simultaneously transfected siRNA into Hep3B and HCCLM3 cells, selecting the highly most effective siRNA-PPP1R16A#1 for subsequent experiments (Fig. 11B and C). CCK8 experiments indicated that knockdown of PPP1R16A suppressed the proliferation capacity ability of Hep3B and HCCLM3 cells (Fig. 11D). Meanwhile, scratch test and Transwell experiments demonstrated that knockdown of PPP1R16A inhibited the migration and invasion abilities of HCCLM3 and Hep3B cells. These observations suggested that PPP1R16A is a positive regulator of HCC cells (Fig. 11E and F).

## Discussion

Hepatocellular carcinoma (HCC) is a type of tumor with poor prognosis, exhibiting a high incidence rate and mortality rate globally. It is a highly refractory disease, despite advancements in surgical and systemic treatments, the prognosis for HCC remains unsatisfactory. Early diagnosis and treatment can improve the survival rate of HCC patients. It is imperative to detect and identify the progression characteristics of the tumor at an early stage. Therefore, developing new treatment techniques and identifying new therapeutic targets is of critical importance.Currently, endoplasmic reticulum stress has garnered widespread attention from various cancer researchers. During endoplasmic reticulum stress, when cells are overly stimulated, the unfolded protein response (UPR) is triggered. This response influences the regulation of the endoplasmic reticulum (ER) balance through three distinct receptors: IRE1α, PERK, and ATF6. Research has shown that the activation of ERS affects tumor cell proliferation, invasion, metastasis, and promotes rapid tumor progression [24, 25].However, the biological mechanisms underlying ERSRGs remain unclear, and their impact on HCC warrants further exploration。.

In this study, we conducted a comprehensive analysis of transcriptomic data between liver cancer and normal tissues. We identified differentially expressed genes (DEGs) between liver cancer and normal tissues, as well as the functions and molecular pathway enrichment of these DEGs. Based on the expression profile analysis of the training set, six endoplasmic reticulum stress-related genes (ERSRGs) were identified using the Random Forest (RF) and Support Vector Machine (SVM) algorithms. Subsequently, an artificial neural network (ANN) prediction model was constructed and demonstrated effective predictive performance. This model was further validated on three independent test sets, confirming its superior predictive capability. We also conducted an in-depth study on the association and function of these genes in tumorigenesis and immunomodulation.

The current (ERSRGs) encompass six potential genes (SRPX, THBS4, CTH, PPP1R16A, CLGN, and THBS1). In fact, previous studies have elucidated the significant roles of some of these genes in various tumors. Cystathionine γ-lyase, encoded by the CTH gene, plays a crucial role in the cysteine sulfur metabolism pathway. It catalyzes the generation of hydrogen sulfide (H_2_S), L-cysteine, α-ketobutyrate, and ammonia [26]. Several studies have indicated that aberrant activation of the CTH/H_2_S signaling pathway is closely linked to the occurrence and progression of HCC [27]. X-rays activate the p38 mitogen-activated protein kinase, which in turn activates the CTH/H_2_S signaling pathway, inducing epithelial-mesenchymal transition and promoting invasion of liver cancer cells [28]. Recent research has also reported that FOXC1, by regulating CTH, inhibits cysteine metabolism, increases reactive oxygen species levels, and promotes tumorigenesis. Overexpression of CTH significantly inhibits the proliferation, invasion, and metastasis of liver cancer cells induced by FOXC1 [29]. In contrast, CTH presents a potential therapeutic target when normally regulated in contrast to FOXC1. Furthermore, Sushi repeat-containing protein X-linked (SRPX) has been identified as a potential therapeutic target in HCC treatment. SRPX has been identified through mRNA expression network analysis, and has been shown to suppress cancer cell stemness [30]. SRPX also regulates the migration and invasion of ovarian cancer through the Ras homolog family member A signaling pathway [31]. Thrombospondin-1 (THBS1), known for inhibiting angiogenesis, has been studied for its potential as a therapeutic target [32]. THBS1 promotes the progression and development of various cancers by regulating angiogenesis and tumor vascular perfusion [33]. Additionally, THBS1 modulates innate and adaptive immune cells through the CD47 signaling molecules, thereby restricting anti-tumor immunity [34]. Overexpression of THBS4 promotes the proliferation and migration of liver cancer cells, participates in the regulation of epithelial-mesenchymal transition progression and interacts with members of the integrin family to modulate the FAK/PI3K/AKT pathway [35]. The miR-142 is highly correlated with THBS4 overexpression in HCC tissue samples, by regulating THBS4 expression in HCC cells [36], PPP1R16A encoded the membrane-associated subunit of protein phosphatase 1 which is located on the plasma membrane as a CAR-binding protein [37]. The area under the ROC curve for PPP1R6A in global and initial-stage tumors was 0.82 and 0.76, respectively, showing excellent sensitivity and specificity to define the diagnosis likelihood of endometrial carcinoma [38]. However, the role of PPP1R6A in HCC diagnosis and prognosis is rarely known and requires further exploration. A recent study reported that upregulation of CLGN in HCC is significantly related to poor prognosis, especially in advanced stages which might be regulated by miR-194-3p, thus providing a potentially therapeutic target and prognosis predictor in HCC [39].

In cellular communication analysis, we found that the expression levels of these ERSRGs were closely associated with immune cell infiltration and the activity of immune-related pathways. Single-cell sequencing revealed that the high expression of PPP1R16A in the liver parenchyma may be a trigger for high-copy mutations. Given that MIF acted as a macrophage stimulator, we speculate from cell communication results that PPP1R16A cells may promote macrophage aggregation through the MIF pathway, which inducing M2 polarization of liver cancer cells. Recent study suggests that novel ERSRGs signature could an independent prognostic factor for HCC [40]. ERS regulate immune levels by regulating myeloid cells, mainly macrophages which is related to tumor evasion of the immune response, and chemoresistance. ER-stressed HCC cells release exosomes, upregulate the expression of PD-L1 in macrophages, and consequently suppresses T-cell function. A higher density of infiltrated macrophages in the liver has been observed to be associated with enhanced tumor aggressiveness and unfavorable prognosis among patients with HCC [11].

In recent years, immune checkpoint inhibitors (ICIs) as a new therapeutic approach targeting T cells regulatory pathways, have much attention [41, 42], and have great prospects in the field of anti-tumor therapy. Through the analysis of ssGSEA results, we found that THBS1 and SRPX showed significant positive correlations with immune cell infiltration, neutrophils, helper T cells, TILs, CCR, and inflammatory response-related pathways. In contrast, THBS4, PPP1R16A, and CLGN exhibited significant negative correlations with immune cell infiltration, neutrophils, helper T cells, TILs, CCR, immune checkpoints, T cell co-inhibition, and other immune-related pathways. These findings suggest that ERSRGs is closely related to the immune status of liver cancer, and offered a new research direction for the combination targeting of ERSRGs and ICIs in the treatment of liver cancer. Through combination therapy, there is potential to enhance anti-tumor immune responses and improve the prognosis of liver cancer.

We also found in cellular communication analysis that, simultaneously, fibroblasts, as essential factors promoting tumor metastasis, may reshape the tumor microenvironment by enhancing the collagen pathway and promoting collagen deposition to affect the function of PPP1R16A cells. These results further indicate that PPP1R16A may influence the prognosis of HCC by regulating the tumor immune microenvironment. Additionally, our experimental results suggest that knocking out PPP1R16A can inhibit the proliferation, invasion, and migration capabilities of HCC cells, indicating that PPP1R16A may be a crucial tumor-promoting factor. Cancer-associated fibroblasts produce collagen and change the extracellular matrix, which is an important mechanism of tumor metastasis. Modulating targeting specific signaling molecules responsible for crosstalk between Cancer‐associated fibroblasts and tumor cells is considered a promising approach to modulating HCC metastasis [43, 44]. Our study indicated that PPP1R16A may be one of such potential targets.

However, it is important to note that there are some limitations that need further addressing and in-depth exploration. Firstly, Considering the bioinformatics analysis based on public cancer databases, it is crucial to further validate the diagnostic and predictive performance of ERSRG markers in large-scale and prospective clinical trials and assess their potential clinical applications. This will contribute to ensuring the reliability and reproducibility of the analysis results and provide a more solid foundation for the clinical application of ERSRGs in liver cancer patients. Secondly, Despite some cell experimental validation was involved, providing support for preliminary findings, further in vivo and in vitro experiments are needed to thoroughly investigate the functions of ERSRGs in HCC. This expanded experimental research will contribute to a more comprehensive and in-depth understanding of the exact mechanisms of action of these genes in the development and progression of HCC, and contribute to a more comprehensive understanding of the potential efficacy and mechanisms of ERSRGs in combination with ICIs in liver cancer. Therefore, future research directions should include broader experimental designs to more comprehensively and systematically reveal the role of ERSRGs in the biology of HCC biology.

## Conclusions

In this study, the researchers integrated the six identified ERSRGs into an ANN prediction model based on RF and SVM algorithms. Furthermore, we further investigated the biological mechanisms, immune regulation, and genomic mutations associated of these six ERSRGs in the diagnosis of liver cancer. The comprehensive analysis of ERSRGs provides a powerful tool for the prognosis prediction and personalized treatment of liver cancer patients. The feature model based on ERSRGs holds promise as a crucial prediction and therapeutic decision support system in the field of liver cancer research.

## Data Availability

All data generated or analyzed during this study are included in this published article.

## Abbreviations

HCC: Hepatocellular Carcinoma
ANN: Artificial Neural Network
ERS: Endoplasmic Reticulum Stress
PPP1R16A: Protein Phosphatase 1 Regulatory Subunit 16 A
AUC: Area Under the Curve
ERSRG: Endoplasmic Reticulum Stress Related Gene
UPR: Unfolded Protein Respons
DEGs: Differential Expressed Genes
GEO: Gene Expression Omnibus
GO: Gene Ontology
KEGG: Kyoto Encyclopedia of Genes and Genomes
GSVA: Gene Set Variance Analysis
ROC: Receiver Operating Characteristic
UMAP: Uniform Manifold Approximation and Projection
RF: Random Forest
SVM: Support Vector Machine
GEPIA: Gene Expression Profiling Interactive Analysis
PCs: Principal Components
SRPX: Sushi Repeat-Containing Protein X-linked
THBS1: Thrombospondin-1
MIF: Macrophage Migration Inhibitory Factor
ICIs: Immune Checkpoint Inhibitors
